# DASES: a database of alternative splicing for esophageal squamous cell carcinoma

**DOI:** 10.3389/fgene.2023.1237167

**Published:** 2023-11-10

**Authors:** Yilong Chen, Yalan Kuang, Siyuan Luan, Yongsan Yang, Zhiye Ying, Chunyang Li, Jinhang Gao, Yong Yuan, Haopeng Yu

**Affiliations:** ^1^ Department of Thoracic Surgery and West China Biomedical Big Data Center, West China Hospital, Sichuan University, Chengdu, China; ^2^ Med-X Center for Informatics, Sichuan University, Chengdu, China; ^3^ Department of Gastroenterology, West China Hospital, Sichuan University, Chengdu, China; ^4^ Laboratory of Gastroenterology and Hepatology, State Key Laboratory of Biotherapy, West China Hospital, Sichuan University, Chengdu, China

**Keywords:** esophageal squamous cell carcinoma, alternative splicing, database, novel lncRNA, isoform

## Abstract

Esophageal carcinoma ranks as the sixth leading cause of cancer-related mortality globally, with esophageal squamous cell carcinoma (ESCC) being particularly prevalent among Asian populations. Alternative splicing (AS) plays a pivotal role in ESCC development and progression by generating diverse transcript isoforms. However, the current landscape lacks a specialized database focusing on alternative splicing events (ASEs) derived from a large number of ESCC cases. Additionally, most existing AS databases overlook the contribution of long non-coding RNAs (lncRNAs) in ESCC molecular mechanisms, predominantly focusing on mRNA-based ASE identification. To address these limitations, we deployed DASES (http://www.hxdsjzx.cn/DASES). Employing a combination of publicly available and in-house ESCC RNA-seq datasets, our extensive analysis of 346 samples, with 93% being paired tumor and adjacent non-tumor tissues, led to the identification of 257 novel lncRNAs in esophageal squamous cell carcinoma. Leveraging a paired comparison of tumor and adjacent normal tissues, DASES identified 59,094 ASEs that may be associated with ESCC. DASES fills a critical gap by providing comprehensive insights into ASEs in ESCC, encompassing lncRNAs and mRNA, thus facilitating a deeper understanding of ESCC molecular mechanisms and serving as a valuable resource for ESCC research communities.

## 1 Introduction

Esophageal carcinoma (EC), a type of malignant tumor affecting the esophagus, is a major global health concern with an estimated annual incidence of over 600,000 and mortality of over 500,000, making it the seventh most common malignant tumor and the sixth leading cause of cancer-related death globally (Sung et al., 2021). There are significant regional differences in the incidence of EC, which can be divided into esophageal squamous cell carcinoma (ESCC) and esophageal adenocarcinoma (EAC), according to the pathological type, with nearly 79% of ESCC occurring in Asian countries (Morgan et al., 2022). Although the incidence of ESCC has shown a decreasing trend in certain countries (Liang et al., 2017), ESCC continues to be a pressing public health issue on account of its increased fatality rate (Abnet et al., 2018). Majority of ESCC patients present at an advanced stage during medical consultation, and conventional surgical interventions often exhibit suboptimal effectiveness or even fail to achieve a radical resection in some cases (He et al., 2022; Pape et al., 2022), with a 5-year survival rate of less than 30% (Allemani et al., 2018). As tumor molecular biology and immune escape mechanisms are more thoroughly studied, a growing number of targeted and immune drugs are being investigated as potential treatments to prolong the survival time of patients with ESCC (Kojima et al., 2020; Costoya and Arce, 2023).

Alternative splicing (AS) is a post-transcriptional regulatory process that generates various RNA isoforms by employing diverse splicing patterns, thereby playing a pivotal role in regulating protein production, especially during developmental and differentiation processes (Yang et al., 2016; Bonnal et al., 2020). When AS is not properly regulated, it can result in the production of oncogenic isoforms, which can contribute to the growth and progression of tumors (Zhang et al., 2021). ESCC patients exhibit a high frequency of alternative splicing events (ASEs), which are associated with tumor initiation, progression, invasion, and immune evasion (Dlamini et al., 2021; Wu et al., 2021). Meanwhile, AS has potential importance in the treatment of ESCC, and several studies suggest that intervention in AS can enhance the sensitivity of ESCC cells to chemotherapy drugs (Siegfried and Karni, 2018; Sciarrillo et al., 2020). Additionally, AS has been shown to impact the efficacy of immunotherapy for ESCC by influencing the expression and presentation of tumor antigens, ultimately affecting the recognition and attack of tumor cells by immune cells (Duan et al., 2021; Wu et al., 2021). Thus, AS has important implications and value for a deeper understanding of the molecular mechanisms of ESCC and the development of therapeutic and immunotherapeutic strategies.

Currently, several databases are available that encompass ASEs, including some that cover ESCC, such as TCGASpliceSeq (Deng et al., 2021) and OncoSplicing (Zhang et al., 2022), developed based on data from The Cancer Genome Atlas (TCGA) (Tomczak et al., 2015). However, despite the inclusion of multiple cancer types, the number of ESCC cases in these databases is limited, with only 96 cases available (Yang et al., 2023). Furthermore, most of these databases primarily rely on oligo dT and poly A sequencing techniques, focusing on AS identification in protein-coding genes, with limited attention given to AS events involving long non-coding RNAs (lncRNAs). In contrast, although ESCC-specific databases, such as ESCCdb (Yang et al., 2023) and CCGD-ESCC (Peng et al., 2018), encompass a larger number of cases, they lack the specific annotation of ASEs. Considering the significant relationship between lncRNA expression and ESCC development and progression (Li et al., 2019b; Razavi and Ghorbian, 2019; Sadeghpour and Ghorbian, 2019; Aalijahan and Ghorbian, 2020; Liu et al., 2020; Ghasemzadeh and Ghorbian, 2023) and the absence of specialized AS-related databases for ESCC, we developed the Database of Alternative Splicing for Esophageal Squamous cell carcinoma (DASES) (http://www.hxdsjzx.cn/DASES), which utilizes two main sets of data. The first set consists of our in-house total transcriptome sequencing data, derived from ESCC patients at the West China Hospital of Sichuan University. The second set is total transcriptome sequencing data from 11 published projects related to ESCC. Through the integration of known transcripts, the identification of novel lncRNAs, and the paired comparison of isoforms between tumor and adjacent normal tissues, DASES provides a comprehensive and precise catalog of ASEs in ESCC, filling a critical gap in the field and offering a valuable resource for ESCC research communities.

## 2 Materials and methods

### 2.1 Data collection

**FIGURE 1:**
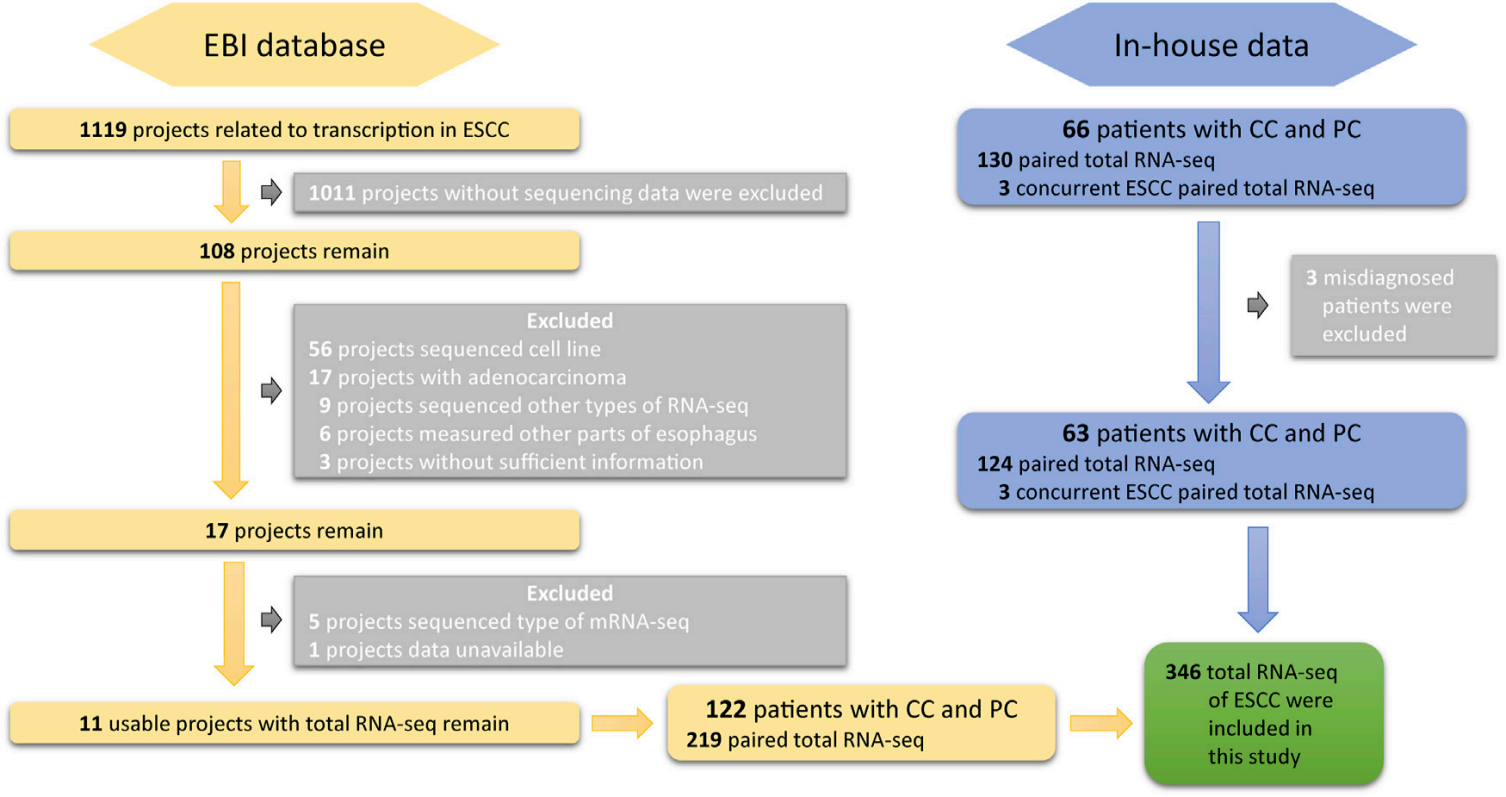
Overview of the screening and inclusion criteria for ESCC patient transcriptome data in the study.

DASES contains raw data from two sources. The first source includes total RNA sequencing data on both tumor and adjacent normal tissues from 63 ESCC patients in West China Hospital of Sichuan University. The second source includes publicly available total RNA sequencing data on ESCC patients from the European Bioinformatics Institute (EBI). To ensure high-quality data, we employed strict search criteria to select suitable samples from EBI (Figure 1): 1) the samples were obtained from human ESCC tissues; 2) the data included RNA sequencing; and 3) the data had sufficient information available. We excluded the cell line RNA-seq data, RNA-seq data from esophageal adenocarcinoma or other parts of the esophagus, and any data without sufficient information. It is essential to emphasize that the included datasets were not specifically targeted or enriched for circular RNA (circRNA) or small RNA during the sequencing and library preparation processes.

### 2.2 Data quality control and lncRNA identification

**FIGURE 2:**
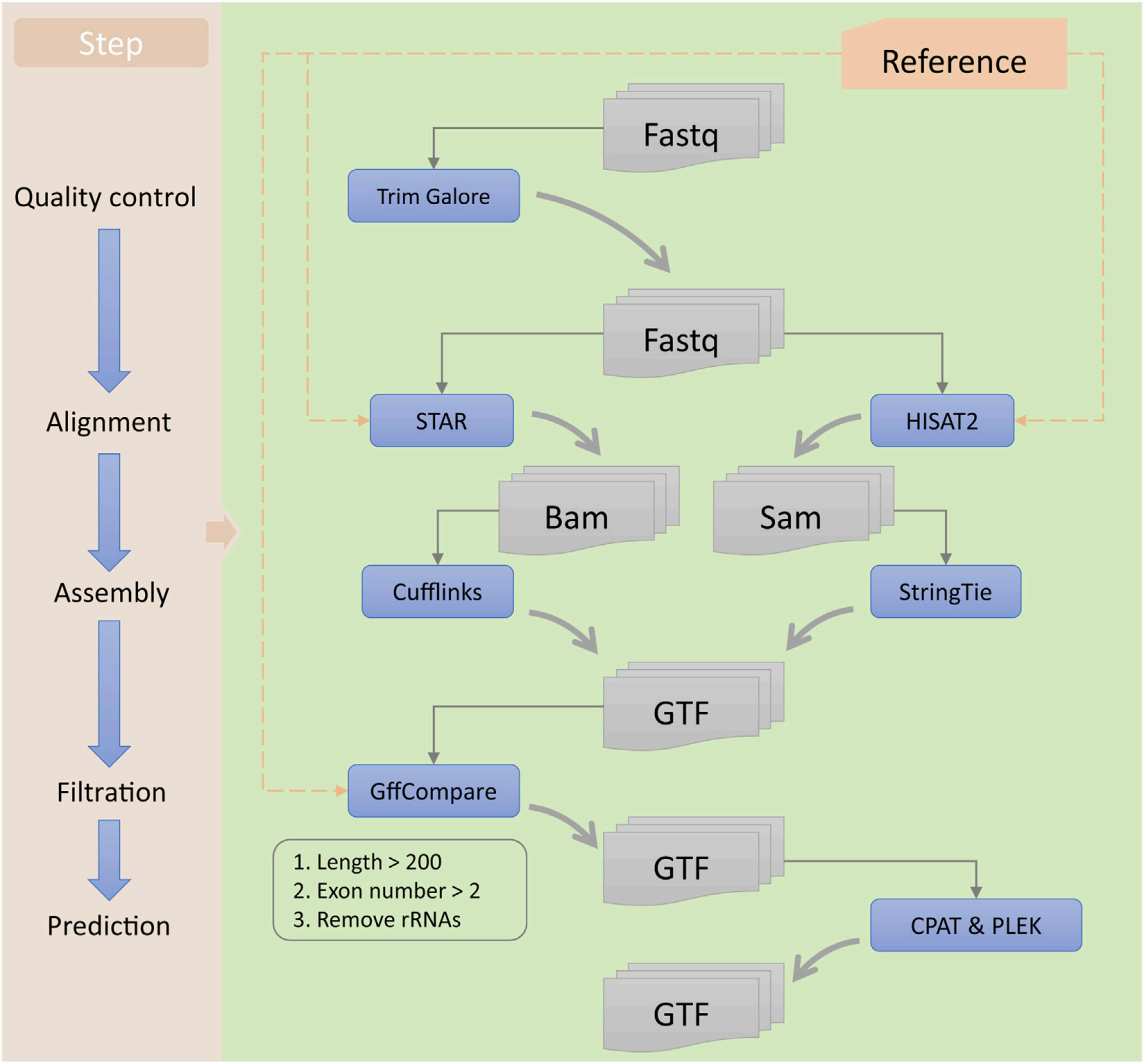
Workflow for the identification of lncRNAs. The left panel represents each step of the identification process, while the right panel includes the tools used and the types of input and output files for each step.

In this study, we used a series of bioinformatics tools to identify potential lncRNAs and mRNAs associated with ESCC (Figure 2). First, the raw reads obtained from RNA-seq were subjected to quality control using Trim Galore software (version 0.6.4; https://www.bioinformatics.babraham.ac.uk/projects/trim_galore) to obtain clean reads. Then, we used STAR (version 2.7.3a) (Dobin et al., 2013) and HISAT2 software programs (version 2.2.1) (Kim et al., 2019) for sequence alignment of the clean data, resulting in the generation of BAM and SAM files for each sample, respectively. Next, we used Cufflinks (version 2.2.0) (Trapnell et al., 2010) and StringTie software programs (version 2.1.4) (Pertea et al., 2015) to assemble the BAM and SAM files, respectively, generating GTF files for each sample. We then used StringTie software to merge all the assembled GTF files, obtaining a preliminary merged GTF file. Subsequently, we utilized GffCompare software (version 0.12.2) (Pertea and Pertea, 2020) and reference transcripts to identify potential lncRNAs and mRNAs. We selected transcripts with class code “i” or “u” as potential lncRNA candidates and those with class code “=,” “c,” or “j” as mRNA candidates. Finally, we predicted the lncRNA candidates using CPAT (version 3.0.2) (Wang et al., 2013) and PLEK software programs (version 1.2) (Li et al., 2014), and selected those predicted as non-coding RNAs by both tools as lncRNA candidates. We merged the lncRNA and mRNA candidates to generate a comprehensive GTF file containing all potential lncRNA and mRNA candidates associated with ESCC. All the analyses were conducted using the human genome hg38 (release 84) reference provided by Ensembl (https://ensembl.org/Homo_sapiens/Info/Index).

### 2.3 Alternative splicing event identification

**FIGURE 3:**
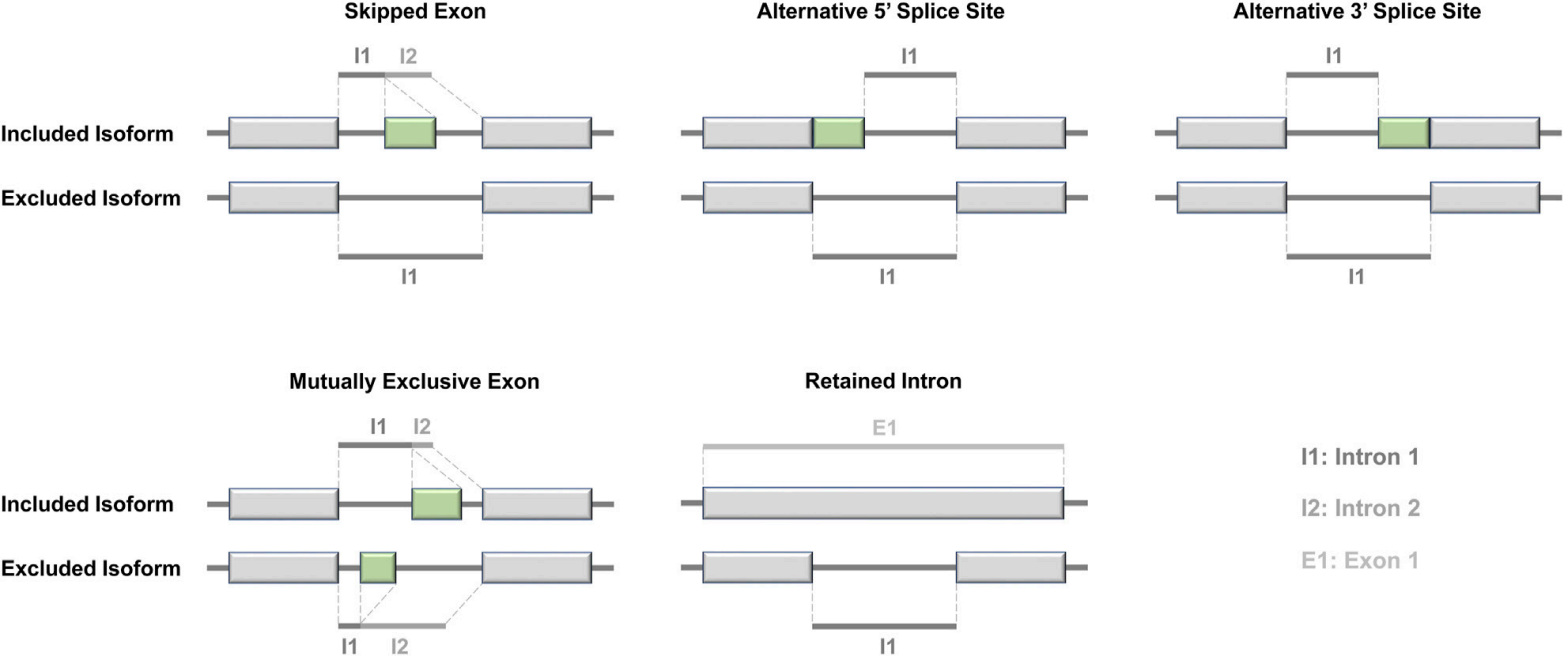
Classification of alternative splicing events.

ASEs can manifest in different ways, including skipping an exon (SE), including or excluding a mutually exclusive exon (MXE), using alternative 5′ or 3′ splice sites (A5SS or A3SS), or retaining an intron (RI). To determine the occurrence of ASEs, we compared two different transcript isoforms derived from the same gene. Specifically, we performed paired comparisons between tumor and adjacent normal groups. In these comparisons, we assigned the term “included isoform” to the isoform containing exons when comparing two transcripts. Conversely, the isoform lacking exons was referred to as the “excluded isoform.” The designation of the “included isoform” was based on having a shorter intron length, whereas the “excluded isoform” had a longer intron length (Figure 3). By comparing the splice junctions and exon–intron boundaries between these two isoforms, we identified and quantified the specific ASEs present in the transcriptome.

To comprehensively identify ASEs associated with ESCC, we employed rMATS software (version 3.1.0) (Shen et al., 2014) with a stringent splicing difference cutoff of 0.0001. Given the publicly available data literature reports, which indicated that all the whole-transcriptome data utilized dUTP-based library construction techniques, we considered the fr-firststrand library type during the analysis of aligned reads in BAM format. By comparing exon inclusion levels between tumor and adjacent normal groups, we detected differential ESCC-related ASEs. We only retained ASEs with a percent spliced in (PSI) (Katz et al., 2010) value greater than 0 and that were present in at least two samples. The results of splicing with only reads that span splicing junction based on GTF files were selected as the ESCC-related ASEs. Furthermore, to establish the coordinates of ASEs, we considered that each ASE comprises two transcript isoforms, with each isoform potentially containing 0–2 introns. In order to define the boundaries of ASE, we determined the minimum coordinate of the intron within the event as the starting coordinate and the maximum coordinate of the intron as the ending coordinate.

### 2.4 Expression quantitative analysis

For quantitative analysis of the RNA-seq data, we employed the merged GTF file as the reference annotation. The BAM files, generated from the alignment step, were subjected to subsequent analysis using Cuffnorm (version 2.2.0), which is a part of Cufflinks software. This tool allowed us to estimate the expression levels of individual isoforms, providing fragments per kilobase of transcript per million mapped reads (FPKM) values.

### 2.5 Potential affected the protein domain by alternative splicing

To assess the potential overlap between ASEs and protein domains, we adopted a conservative approach. We focused only on the known protein domains that intersected with ASEs, disregarding the predictions from various tools. Initially, we retrieved protein domain information from the Ensembl database, specifically focusing on the hg38 version of protein domain annotations (release 109), which includes InterPro coordinates, associated transcripts, and corresponding genes. We then mapped the InterPro coordinates onto genomic coordinates using appropriate alignment algorithms as follows:

$${Start}_{genomic}={Start}_{CDS}+3\times {Start}_{InterPro}-3,$$

$${End}_{genomic}={Start}_{CDS}+3\times {End}_{InterPro}-1,$$

where ${Start}_{genomic}$ and ${End}_{genomic}$ represent the start and end sites of the protein domain on the genome, respectively, ${Start}_{CDS}$ refers to the first CDS start site of the corresponding transcript, and ${Start}_{InterPro}$ and ${End}_{InterPro}$ indicate the start and end sites of the protein domain on InterPro coordinates, respectively. Subsequently, we scrutinized whether there was any intersection or overlap between the genomic coordinates of protein domains and genomic coordinates of ASEs. In the cases where a protein domain exhibited any intersection or overlap with an ASE, we deemed it as having a significant overlap with the respective ASE.

### 2.6 Deployment of DASES

DASES is readily accessible through its website at http://www.hxdsjzx.cn/DASES, and no registration or login is required for usage. The current version of DASES was deployed utilizing MySQL (version 8.0.18) (http://www.mysql.com) and operates on a Linux-based Aliyun web server. Server-side scripting was implemented using Tomcat (version 8.0) (http://tomcat.apache.org/) and JAVA (version 1.8) (https://www.oracle.com/technetwork/java/index.html), providing the necessary functionality. The user-friendly web interface of DASES was created using Bootstrap (version 3.3.7) (https://v3.bootcss.com) and jQuery (version 2.1.1) (http://jquery.com) for seamless interaction and enhanced user experience. Genomic visualization capabilities were achieved using JBrowse (http://jbrowse.org) and IGV (https://igv.org), while additional visualizations were facilitated by ECharts (https://echarts.apache.org/zh/index.html). The web interface of DASES comprises various modules, including Home, Search, Browse, Genome Browser, Download, and About, ensuring comprehensive and intuitive access to the platform’s features and information.

## 3 Results

### 3.1 Data and database overview

**FIGURE 4:**
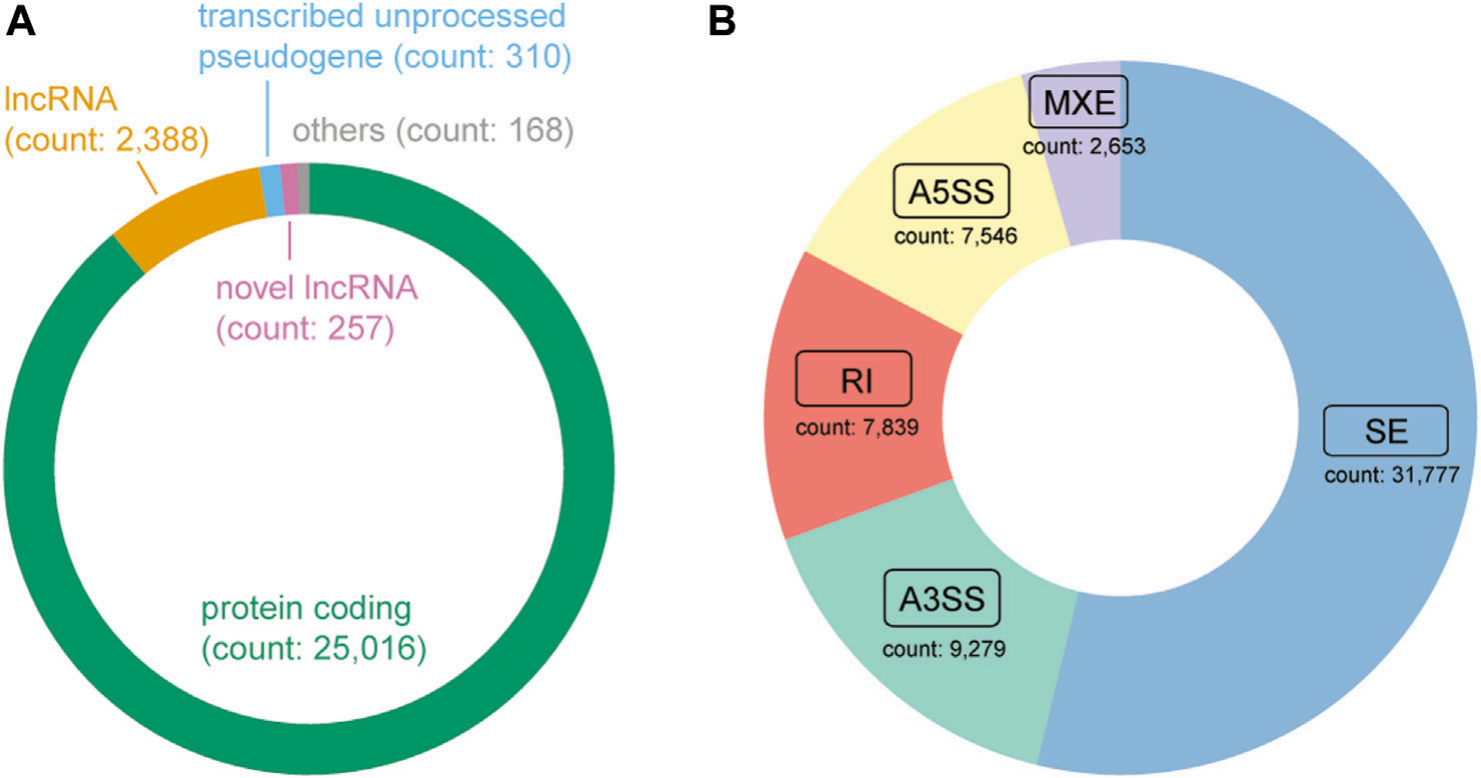
Composition of the gene type and alternative splicing event type in DASES. **(A)** Distribution of gene biotypes, where “Others” comprise a combination of transcribed unitary pseudogene, transcribed processed pseudogene, miRNA, TEC, unprocessed pseudogene, and IG C gene. **(B)** Distribution of alternative splicing event types.

Following the application of quality control measures, a total of 14 patients, corresponding to 28 samples, were eliminated from the dataset. Currently, DASES encompasses data from the in-house study and 11 publicly available studies (Table 1), comprising a total of 346 samples, with 185 distinct ESCC patients represented. We identified 257 novel lncRNAs (Figure 4A) and a total of 59,094 ASEs by using a tumor versus adjacent normal strategy, with 31,777 belonging to SE, 7,546 utilizing A5SS, 9,279 utilizing A3SS, 2,653 involving MXE, and 7,839 RI (Figure 4B).

**TABLE 1:** Information on whole-transcriptome data in ESCC patients from one in-house study and 11 publicly available studies.

Study accession	Number of samples (tumor:adjacent)^a^	Geographic position	Layout	Sequencing library	Data accession
PRJCA017448	64:63	China	Paired	dUTP	https://ngdc.cncb.ac.cn/search/?dbId=hra&q=PRJCA017448
PRJNA793370	3:3	China	Paired	dUTP	https://www.ncbi.nlm.nih.gov/bioproject/793370
PRJNA843947	6:6	China	Paired	dUTP	https://www.ncbi.nlm.nih.gov/bioproject/843947
PRJNA784605	4:4	China	Paired	dUTP	https://www.ncbi.nlm.nih.gov/bioproject/784605
PRJNA665149	18:18	China	Paired	dUTP	https://www.ncbi.nlm.nih.gov/bioproject/665149
PRJNA689307	8:8	China	Paired	dUTP	https://www.ncbi.nlm.nih.gov/bioproject/689307
PRJNA629358	10:10	China	Paired	dUTP	https://www.ncbi.nlm.nih.gov/bioproject/629358
PRJNA594797	3:3	China	Paired	dUTP	https://www.ncbi.nlm.nih.gov/bioproject/594797
PRJNA608223	0:25	Kazakhstan	Paired	dUTP	https://www.ncbi.nlm.nih.gov/bioproject/608223
PRJNA533799	23:23	Korea	Paired	dUTP	https://www.ncbi.nlm.nih.gov/bioproject/533799
PRJNA435587	7:7	China	Paired	dUTP	https://www.ncbi.nlm.nih.gov/bioproject/435587
PRJNA298963	15:15	China	Paired	dUTP	https://www.ncbi.nlm.nih.gov/bioproject/298963

^a^ The number of samples on tumor tissues versus the number of samples on adjacent normal tissues for ESCC patients in each study.

**FIGURE 5:**
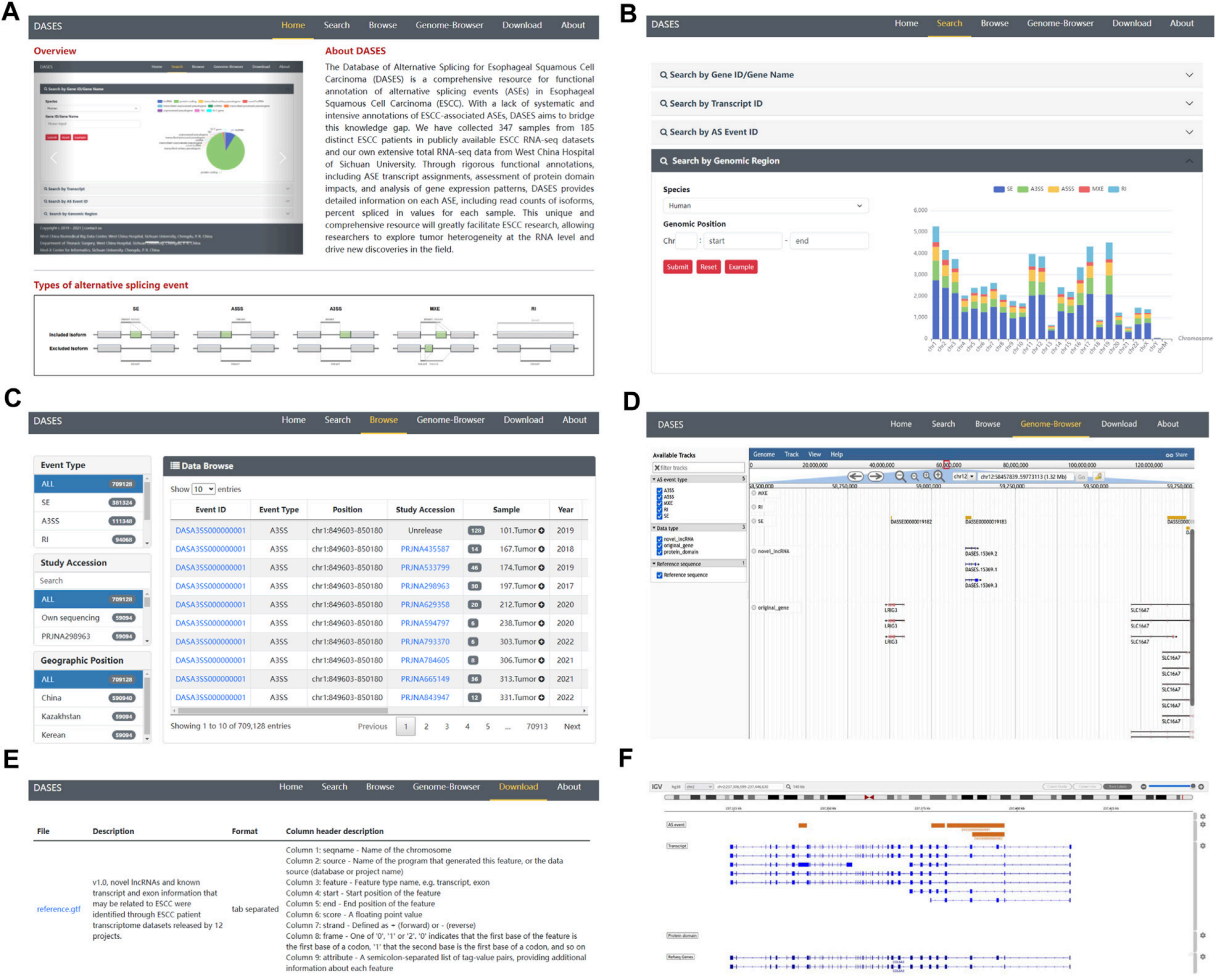
Overview of DASES module interfaces. **(A)** Home interface, **(B)** Search interface, **(C)** Browse interface, **(D)** Genome Browser, **(E)** Download interface, and **(F)** Genome Browser after performing a search.

To facilitate easy access and utilization of the database, we designed a user-friendly web interface featuring various modules. The Home page provides users with a concise overview of DASES, accompanied by illustrative diagrams showcasing the five major types of ASEs (Figure 5A). The Search page offers four different search options, namely, gene, transcript, ASE ID, and genomic region, facilitating easy and efficient data retrieval (Figure 5B). The Browse page provides a comprehensive list of all ASE IDs, allowing users to narrow down their queries by applying filters based on the ASE type or study name (Figure 5C). The Genome Browser page enables users to visualize the genomic regions associated with ASEs (Figure 5D). The Download page offers convenient access to essential files, including processed GTF file and ASE-related data, which can be downloaded for further analysis (Figure 5E). Lastly, the About page serves as a valuable resource, providing a detailed pipeline overview of the entire database, along with comprehensive explanations of important interface features, including headers and abbreviations for primary tables, enabling users to fully comprehend and navigate the database with ease.

### 3.2 Diversified search strategies

In DASES, we present a comprehensive search system comprising four dimensions (Figure 5B). The first dimension allows users to conduct searches based on the gene ID or gene name, thereby retrieving pertinent gene information alongside details concerning gene-associated ASEs. By employing the second dimension, users can search using the transcript ID, obtaining transcript-specific information, expression levels across samples, and insights into transcript-associated ASEs. The third dimension facilitates searches based on the ASE ID, yielding ASE-related details, including the exon junction count (EJC), intron junction count (IJC), and PSI values. Finally, the fourth dimension empowers users to search by genomic coordinates, resulting in the retrieval of ASE information specific to designated genomic loci.

### 3.3 Genome Browser visualization

The Genome Browser in DASES comprises two distinct sections. The first section facilitates the visualization of all ASEs (Figure 5D). Within the Genome Browser page, users can utilize diverse tracks to filter ASEs based on specific criteria, including ASE types and chromosome numbers. They also have the option to display or conceal tracks associated with ASEs, transcripts, genes, and protein domains. The second section is accessible via the Search page (Figure 5F). When users conduct searches for genes, transcripts, or specific ASEs, the pertinent information is presented visually on the Genome Browser. This seamless integration of search results with the Genome Browser offers users a contextual perspective on the genomic location of these elements.

### 3.4 Significant association between ESCC TNM staging and ASE frequency

ASEs have been closely linked to tumorigenesis and cancer progression. To investigate whether the frequency of ASEs exhibits an association with TNM staging in ESCC, we conducted a comprehensive analysis using data from DASES. As shown in Supplementary Figures S1A, D, both the frequency of genes undergoing alternative splicing (AS-gene frequency) and the frequency of ASEs in genes exhibiting AS (ASE frequency) exhibited a substantial increase within ESCC tissues when compared to adjacent normal tissues. Furthermore, our analysis unveiled a significant trend in the correlation between ESCC TNM staging and AS-gene frequency (Supplementary Figures S1B, C), as well as ASE frequency (Supplementary Figures S1E, F). These findings underscore a compelling association between ESCC TNM staging and the frequency of ASEs, suggesting their potential relevance in the context of ESCC progression. The source of DASES facilitates the exploration of these intricate relationships, providing a valuable platform for future research in this field.

### 3.5 Consistency with literature findings for *COL6A3* in DASES

**FIGURE 6:**
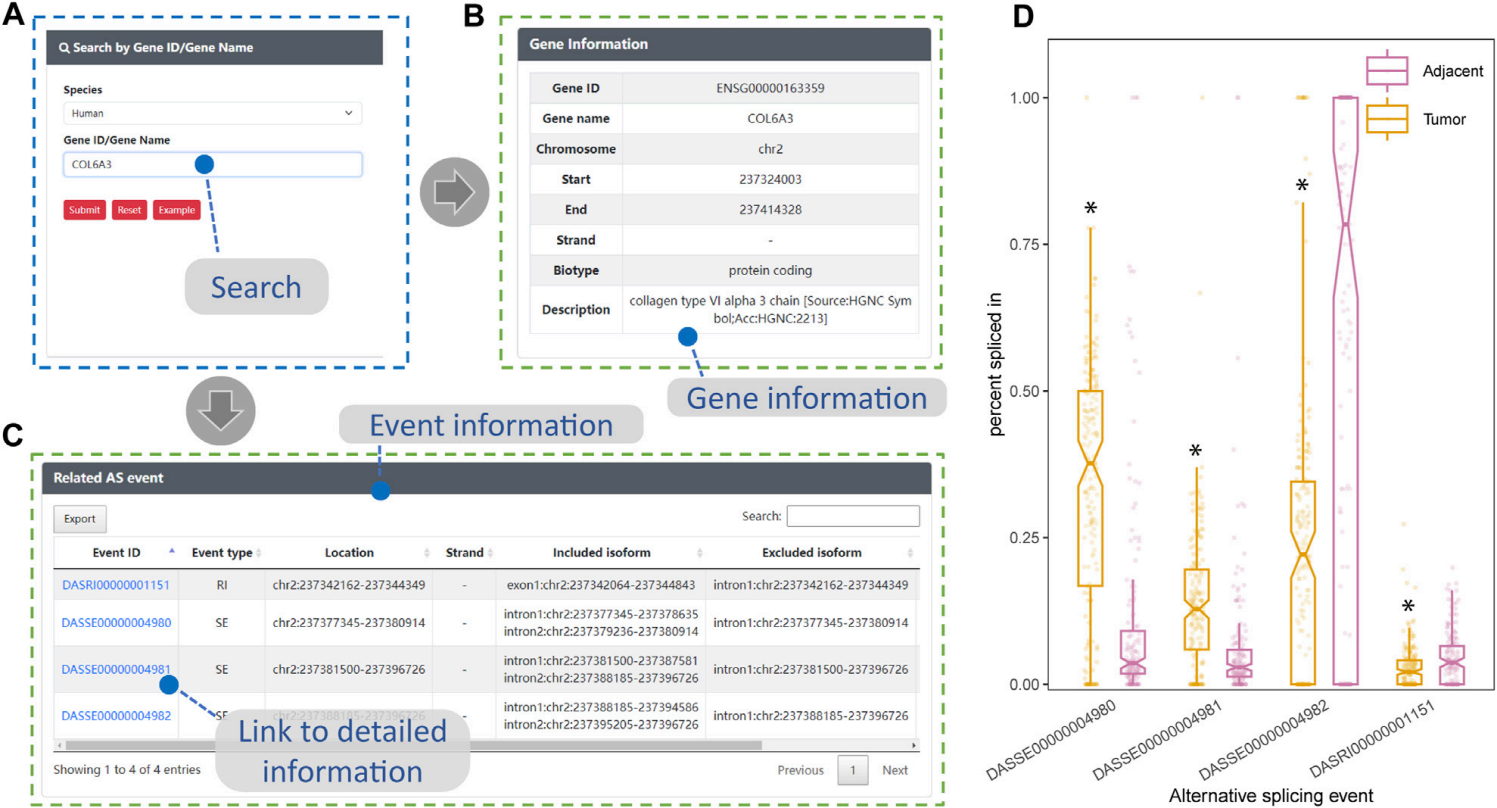
Consistency between DASES results and literature findings for *COL6A3*. This figure showcases the validation process of DASES by employing an example search for the highly expressed *COL6A3* in esophageal tissue. **(A)** Process of searching for *COL6A3* using the search interface. The search results, including relevant descriptions of *COL6A3* in **(B)** and detailed information on the identified alternative splicing events that are related to *COL6A3* in **(C)**. Discovery of associated alternative splicing events that are consistent with literature reports (Ding et al., 2021). **(D)** Disparity in percent spliced in values for these four alternative splicing events between the tumor and adjacent normal tissue groups, as determined by the Mann–Whitney test. *p-value < 0.05.

The expression of *COL6A3* in both bulk esophageal tissue and single esophageal tissue samples exhibited a relatively high level, as evidenced by data obtained from the GTEx website (https://gtexportal.org/home/). Utilizing the search interface of DASES, we specifically queried *COL6A3* (Figures 6A, B), leading to the identification of four ASEs, i.e., three SE events and one RI event (Figure 6C). Notably, our findings closely align with the observations reported by Ding, who identified three SE-type ASEs of *COL6A3* from 11 samples in their study of ESCC tissues (Ding et al., 2021). Intriguingly, in addition to the ASEs reported by Ding, we discovered an additional RI-type ASE, “DASRI00000001151” (chr2: 237342162–237344349), which was not addressed by Ding. This discrepancy could be attributed to our larger sample size, which enabled us to identify more *COL6A3*-related ASEs. Importantly, we observed statistically significant differences in the PSI values of these four ASEs between the tumor and adjacent normal tissue groups, further highlighting their potential significance in ESCC (Figure 6D). This robust consistency between our findings and those of Ding provides substantial evidence for the reliability of ESCC-related ASEs documented within DASES, thereby reinforcing their validity through corroboration with findings from other literature reports.

## 4 Discussion

In this study, we successfully constructed DASES. By integrating publicly available RNA-seq from ESCC patient tissues, DASES provides a comprehensive resource for the identification and exploration of ASEs potentially associated with ESCC. Moreover, DASES stands out as the first specialized database dedicated to ESCC-associated ASEs, addressing the existing gap in ESCC-specific databases in the field of AS.

DASES employed a tumor versus adjacent normal strategy to identify ESCC-associated ASEs, presenting several notable advantages. First, by comparing samples within the same patient, DASES effectively highlights splicing events that are highly likely to be functionally relevant to the development and progression of ESCC, which could reduce the confounding effects of individual genetic variations or splicing differences that are unrelated to ESCC. This strategy has been demonstrated to be effective in previous studies (Xiong et al., 2015; Kahles et al., 2018). Moreover, in line with other research conclusions, the approach enables the identification of ESCC-specific ASEs that may serve as potential biomarkers or therapeutic targets as they reflect the unique molecular characteristics of ESCC (Kalsotra and Cooper, 2011; Sebestyén et al., 2016). Furthermore, by comparing splicing patterns within the same patient, DASES minimizes inter-individual variations and provides a more robust assessment of the splicing changes specifically related to ESCC, enhancing the reliability of the identified ASEs in DASES. In a word, this approach allows for a more effective, accurate, and reliable characterization of ESCC-related AS.

DASES serves as a comprehensive resource that includes whole-transcriptome data to investigate both known ASEs linked to ESCC and novel lncRNAs, along with their associated ASEs that could potentially be implicated in ESCC. The identification of novel lncRNAs and their associated ASEs in ESCC holds great promise for advancing our understanding of the disease. Several studies have highlighted the importance of lncRNAs in cancer development and progression, including ESCC (Chen et al., 2018). These long non-coding RNAs regulate gene expression, modulate signaling pathways, and contribute to the hallmarks of cancer (Huarte, 2015). Therefore, the incorporation of lncRNA-associated ASEs in DASES provides valuable insights into the regulatory complexity underlying ESCC. Moreover, we offer a comprehensive GTF file that incorporates both the known transcriptome information and the newly discovered lncRNAs in DASES. This resource enables the in-depth exploration of the expression patterns, functional implications, and potential interactions of these newly identified lncRNAs in the context of ESCC.

We recognize that ESCC is a multifactorial disease with various pathogenic genes, including but not limited to *TP53*, *NOTCH1*, *CDKN2A*, and *COL6A3* (Gao et al., 2014; Li et al., 2019b; Liu et al., 2022; Ko et al., 2023). Our Gene Ontology (GO) enrichment analysis of differentially expressed genes highlighted “cell adhesion” as one of the top-ranked GO terms closely linked to cancer, with *COL6A3* being among the genes significantly associated with this GO term. Given these factors, we selected *COL6A3* as a representative gene for demonstrating the utility of DASES. Our analysis of ASEs within *COL6A3* revealed intriguing findings. Although consistent with the study conducted by Ding et al. (2021) on paired ESCC tissues for the most part, our dataset uncovered an additional RI-type ASE within *COL6A3* not reported by Ding et al. (2021). This discrepancy could be attributed to our larger sample size, enabling us to capture more ASEs. This novel finding highlights the value of our database in complementing existing knowledge and uncovering potentially clinically relevant splicing events. It also underscores the significance of leveraging a comprehensive resource like DASES to complement existing studies and expand our knowledge of this complex disease.

It is important to acknowledge that DASES also has certain limitations and areas that can be further improved. First, DASES currently focuses exclusively on ESCC patient tissue data and lacks representation from other species. However, human patient tissue data remain valuable, and future versions will include data from diverse organizations to broaden its scope. Second, DASES primarily relies on whole-transcriptome data, neglecting other sequencing data types. Integrating multiple omics data types can enhance our understanding of ESCC mechanisms. Moreover, in evaluating the impact of ASEs on proteins, we only considered instances where ASEs occur directly within protein domains. However, there are other ways in which proteins can be affected, such as a frameshift occurring before a protein domain or ASEs occurring in scaffold regions, which can influence their three-dimensional structure.

## Data Availability

The datasets presented in this study can be found in online repositories. The names of the repository/repositories and accession number(s) can be found in the article/Supplementary Material.

## Supplementary material

SUPPLEMENTARY FIGURE S1 Relationship between the frequency of ASEs and ESCC TNM staging. AS-gene frequency denotes the frequency of genes undergoing AS, while the ASE frequency indicates the frequency of ASEs in genes exhibiting AS. **(A,D)** show the statistically significant difference in the AS-gene frequency and ASE frequency between tumor and adjacent normal tissues, as determined by the Mann–Whitney test (**p-*value < 0.05). **(B,C)** demonstrate the statistically significant trends in the AS-gene frequency concerning ESCC T staging and N staging, and **(E,F)** reveal the statistically significant trends in the ASE frequency with respect to ESCC T staging and N staging, as determined by the Cochran–Armitage trend test (^#^ *p-*value < 0.05).

## References

[B1] Aalijahan H. Ghorbian S. (2020). Clinical application of long non-coding RNA-UCA1 as a candidate gene in progression of esophageal cancer. Pathol. Oncol. Res. 26, 1441–1446. 10.1007/s12253-019-00711-3 31414398

[B2] Abnet C. C. Arnold M. Wei W. Q. (2018). Epidemiology of esophageal squamous cell carcinoma. Gastroenterology 154, 360–373. 10.1053/j.gastro.2017.08.023 28823862 PMC5836473

[B3] Allemani C. Matsuda T. Di Carlo V. Harewood R. Matz M. Nikšić M. (2018). Global surveillance of trends in cancer survival 2000-14 (CONCORD-3): analysis of individual records for 37 513 025 patients diagnosed with one of 18 cancers from 322 population-based registries in 71 countries. Lancet 391, 1023–1075. 10.1016/S0140-6736(17)33326-3 29395269 PMC5879496

[B4] Bonnal S. C. López-Oreja I. Valcárcel J. (2020). Roles and mechanisms of alternative splicing in cancer - implications for care. Nat. Rev. Clin. Oncol. 17, 457–474. 10.1038/s41571-020-0350-x 32303702

[B5] Chen T. Chen X. Zhang S. Zhu J. Tang B. Wang A. (2021). The genome sequence archive family: toward explosive data growth and diverse data types. Genomics Proteomics Bioinforma. 19, 578–583. 10.1016/j.gpb.2021.08.001 PMC9039563 34400360

[B6] Chen X. Chen Z. Yu S. Nie F. Yan S. Ma P. (2018). Long noncoding RNA LINC01234 functions as a competing endogenous RNA to regulate CBFB expression by sponging miR-204-5p in gastric cancer. Clin. Cancer Res. 24, 2002–2014. 10.1158/1078-0432.CCR-17-2376 29386218

[B7] Costoya J. A. Arce V. M. (2023). Cancer cells escape the immune system by increasing stemness through epigenetic reprogramming. Cell Mol. Immunol. 20, 6–7. 10.1038/s41423-022-00953-3 36380095 PMC9794797

[B8] Deng Y. Luo H. Yang Z. Liu L. (2021). LncAS2Cancer: a comprehensive database for alternative splicing of lncRNAs across human cancers. Brief. Bioinform 22, bbaa179. 10.1093/bib/bbaa179 32820322

[B9] Ding J. Li C. Cheng Y. Du Z. Wang Q. Tang Z. (2021). Alterations of RNA splicing patterns in esophagus squamous cell carcinoma. Cell Biosci. 11, 36. 10.1186/s13578-021-00546-z 33563334 PMC7871539

[B10] Dlamini Z. Hull R. Mbatha S. Z. Alaouna M. Qiao Y. L. Yu H. (2021). Prognostic alternative splicing signatures in esophageal carcinoma. Cancer Manag. Res. 13, 4509–4527. 10.2147/CMAR.S305464 34113176 PMC8186946

[B11] Dobin A. Davis C. A. Schlesinger F. Drenkow J. Zaleski C. Jha S. (2013). STAR: ultrafast universal RNA-seq aligner. Bioinformatics 29, 15–21. 10.1093/bioinformatics/bts635 23104886 PMC3530905

[B12] Duan Y. Jia Y. Wang J. Liu T. Cheng Z. Sang M. (2021). Long noncoding RNA DGCR5 involves in tumorigenesis of esophageal squamous cell carcinoma via SRSF1-mediated alternative splicing of Mcl-1. Cell Death Dis. 12, 587. 10.1038/s41419-021-03858-7 34099633 PMC8184765

[B13] Gao Y. B. Chen Z. L. Li J. G. Hu X. D. Shi X. J. Sun Z. M. (2014). Genetic landscape of esophageal squamous cell carcinoma. Nat. Genet. 46, 1097–1102. 10.1038/ng.3076 25151357

[B14] Ghasemzadeh S. Ghorbian S. (2023). Investigation of clinical significant utility of LncRNA-linc02389 in patients with esophageal squamous cell carcinoma. J. Kermanshah Univ. Med. Sci. 27, e136290. 10.5812/jkums-136290

[B15] He W. Wang C. Wu L. Wan G. Li B. Han Y. (2022). Tislelizumab plus chemotherapy sequential neoadjuvant therapy for non-cCR patients after neoadjuvant chemoradiotherapy in locally advanced esophageal squamous cell carcinoma (ETNT): an exploratory study. Front. Immunol. 13, 853922. 10.3389/fimmu.2022.853922 35720312 PMC9201912

[B16] Huarte M. (2015). The emerging role of lncRNAs in cancer. Nat. Med. 21, 1253–1261. 10.1038/nm.3981 26540387

[B17] Kahles A. Lehmann K. V. Toussaint N. C. Hüser M. Stark S. G. Sachsenberg T. (2018). Comprehensive analysis of alternative splicing across tumors from 8,705 patients. Cancer Cell 34, 211–224.e6. 10.1016/j.ccell.2018.07.001 30078747 PMC9844097

[B18] Kalsotra A. Cooper T. A. (2011). Functional consequences of developmentally regulated alternative splicing. Nat. Rev. Genet. 12, 715–729. 10.1038/nrg3052 21921927 PMC3321218

[B19] Katz Y. Wang E. T. Airoldi E. M. Burge C. B. (2010). Analysis and design of RNA sequencing experiments for identifying isoform regulation. Nat. Methods 7, 1009–1015. 10.1038/nmeth.1528 21057496 PMC3037023

[B20] Kim D. Paggi J. M. Park C. Bennett C. Salzberg S. L. (2019). Graph-based genome alignment and genotyping with HISAT2 and HISAT-genotype. Nat. Biotechnol. 37, 907–915. 10.1038/s41587-019-0201-4 31375807 PMC7605509

[B21] Ko K. P. Huang Y. Zhang S. Zou G. Kim B. Zhang J. (2023). Key genetic determinants driving esophageal squamous cell carcinoma initiation and immune evasion. Gastroenterology 165, 613–628.e20. 10.1053/j.gastro.2023.05.030 37257519 PMC10527250

[B22] Kojima T. Shah M. A. Muro K. Francois E. Adenis A. Hsu C. H. (2020). Randomized phase III KEYNOTE-181 study of pembrolizumab versus chemotherapy in advanced esophageal cancer. J. Clin. Oncol. 38, 4138–4148. 10.1200/JCO.20.01888 33026938

[B23] Li A. Zhang J. Zhou Z. (2014). PLEK: a tool for predicting long non-coding RNAs and messenger RNAs based on an improved k-mer scheme. BMC Bioinforma. 15, 311. 10.1186/1471-2105-15-311 PMC4177586 25239089

[B24] Li W. Zhang L. Guo B. Deng J. Wu S. Li F. (2019a). Exosomal FMR1-AS1 facilitates maintaining cancer stem-like cell dynamic equilibrium via TLR7/NFκB/c-Myc signaling in female esophageal carcinoma. Mol. Cancer 18, 22. 10.1186/s12943-019-0949-7 30736860 PMC6367809

[B25] Li Y. Sun Y. Yang Q. Wu J. Xiong Z. Li S. (2019b). Variants in COL6A3 gene influence susceptibility to esophageal cancer in the Chinese population. Cancer Genet. 238, 23–30. 10.1016/j.cancergen.2019.07.003 31425922

[B26] Liang H. Fan J. H. Qiao Y. L. (2017). Epidemiology, etiology, and prevention of esophageal squamous cell carcinoma in China. Cancer Biol. Med. 14, 33–41. 10.20892/j.issn.2095-3941.2016.0093 28443201 PMC5365188

[B27] Liu J. Liu Z. X. Wu Q. N. Lu Y. X. Wong C. W. Miao L. (2020). Long noncoding RNA AGPG regulates PFKFB3-mediated tumor glycolytic reprogramming. Nat. Commun. 11, 1507. 10.1038/s41467-020-15112-3 32198345 PMC7083971

[B28] Liu T. Zhao X. Lin Y. Luo Q. Zhang S. Xi Y. (2022). Computational identification of preneoplastic cells displaying high stemness and risk of cancer progression. Cancer Res. 82, 2520–2537. 10.1158/0008-5472.CAN-22-0668 35536873

[B29] Morgan E. Soerjomataram I. Rumgay H. Coleman H. G. Thrift A. P. Vignat J. (2022). The global landscape of esophageal squamous cell carcinoma and esophageal adenocarcinoma incidence and mortality in 2020 and projections to 2040: new estimates from GLOBOCAN 2020. Gastroenterology 163, 649–658.e2. 10.1053/j.gastro.2022.05.054 35671803

[B30] Pape M. Vissers P. de Vos-Geelen J. Hulshof M. Gisbertz S. S. Jeene P. M. (2022). Treatment patterns and survival in advanced unresectable esophageal squamous cell cancer: a population-based study. Cancer Sci. 113, 1038–1046. 10.1111/cas.15262 34986523 PMC8898723

[B31] Partners C. M. a. (2022). Database resources of the national genomics data center, China national center for bioinformation in 2022. Nucleic Acids Res. 50, D27–D38. 10.1093/nar/gkab951 34718731 PMC8728233

[B32] Peng L. Cheng S. Lin Y. Cui Q. Luo Y. Chu J. (2018). CCGD-ESCC: a comprehensive database for genetic variants associated with esophageal squamous cell carcinoma in Chinese population. Genomics Proteomics Bioinforma. 16, 262–268. 10.1016/j.gpb.2018.03.005 PMC6205081 30208340

[B33] Pertea G. Pertea M. (2020). GFF utilities: GffRead and GffCompare. F1000Res 9, ISCB Comm J-304. 10.12688/f1000research.23297.2 PMC7222033 32489650

[B34] Pertea M. Pertea G. M. Antonescu C. M. Chang T. C. Mendell J. T. Salzberg S. L. (2015). StringTie enables improved reconstruction of a transcriptome from RNA-seq reads. Nat. Biotechnol. 33, 290–295. 10.1038/nbt.3122 25690850 PMC4643835

[B35] Razavi M. Ghorbian S. (2019). Up-regulation of long non-coding RNA-PCAT-1 promotes invasion and metastasis in esophageal squamous cell carcinoma. EXCLI J. 18, 422–428. 10.17179/excli2018-1847 31338011 PMC6635722

[B36] Sadeghpour S. Ghorbian S. (2019). Evaluation of the potential clinical prognostic value of lncRNA-BANCR gene in esophageal squamous cell carcinoma. Mol. Biol. Rep. 46, 991–995. 10.1007/s11033-018-4556-2 30552615

[B37] Sciarrillo R. Wojtuszkiewicz A. Assaraf Y. G. Jansen G. Kaspers G. Giovannetti E. (2020). The role of alternative splicing in cancer: from oncogenesis to drug resistance. Drug Resist Updat 53, 100728. 10.1016/j.drup.2020.100728 33070093

[B38] Sebestyén E. Singh B. Miñana B. Pagès A. Mateo F. Pujana M. A. (2016). Large-scale analysis of genome and transcriptome alterations in multiple tumors unveils novel cancer-relevant splicing networks. Genome Res. 26, 732–744. 10.1101/gr.199935.115 27197215 PMC4889968

[B39] Shen S. Park J. W. Lu Z. X. Lin L. Henry M. D. Wu Y. N. (2014). rMATS: robust and flexible detection of differential alternative splicing from replicate RNA-Seq data. Proc. Natl. Acad. Sci. U. S. A. 111, E5593–E5601. 10.1073/pnas.1419161111 25480548 PMC4280593

[B40] Siegfried Z. Karni R. (2018). The role of alternative splicing in cancer drug resistance. Curr. Opin. Genet. Dev. 48, 16–21. 10.1016/j.gde.2017.10.001 29080552

[B41] Sung H. Ferlay J. Siegel R. L. Laversanne M. Soerjomataram I. Jemal A. (2021). Global cancer statistics 2020: GLOBOCAN estimates of incidence and mortality worldwide for 36 cancers in 185 countries. CA Cancer J. Clin. 71, 209–249. 10.3322/caac.21660 33538338

[B42] Tomczak K. Czerwińska P. Wiznerowicz M. (2015). The Cancer Genome Atlas (TCGA): an immeasurable source of knowledge. Contemp. Oncol. Pozn. 19, A68–A77. 10.5114/wo.2014.47136 25691825 PMC4322527

[B43] Trapnell C. Williams B. A. Pertea G. Mortazavi A. Kwan G. van Baren M. J. (2010). Transcript assembly and quantification by RNA-Seq reveals unannotated transcripts and isoform switching during cell differentiation. Nat. Biotechnol. 28, 511–515. 10.1038/nbt.1621 20436464 PMC3146043

[B44] Wang L. Park H. J. Dasari S. Wang S. Kocher J. P. Li W. (2013). CPAT: coding-Potential Assessment Tool using an alignment-free logistic regression model. Nucleic Acids Res. 41, e74. 10.1093/nar/gkt006 23335781 PMC3616698

[B45] Wu Q. Zhang Y. An H. Sun W. Wang R. Liu M. (2021). The landscape and biological relevance of aberrant alternative splicing events in esophageal squamous cell carcinoma. Oncogene 40, 4184–4197. 10.1038/s41388-021-01849-8 34079089

[B46] Xiong H. Y. Alipanahi B. Lee L. J. Bretschneider H. Merico D. Yuen R. K. (2015). RNA splicing. The human splicing code reveals new insights into the genetic determinants of disease. Science 347, 1254806. 10.1126/science.1254806 25525159 PMC4362528

[B47] Yang J. Bi L. Wang C. Wang G. Gou Y. Dong L. (2023). ESCCdb: a comprehensive database and key regulator exploring platform based on cross dataset comparisons for esophageal squamous cell carcinoma. Comput. Struct. Biotechnol. J. 21, 2119–2128. 10.1016/j.csbj.2023.03.026 36968016 PMC10036886

[B48] Yang X. Coulombe-Huntington J. Kang S. Sheynkman G. M. Hao T. Richardson A. (2016). Widespread expansion of protein interaction capabilities by alternative splicing. Cell 164, 805–817. 10.1016/j.cell.2016.01.029 26871637 PMC4882190

[B49] Zhang Y. Qian J. Gu C. Yang Y. (2021). Alternative splicing and cancer: a systematic review. Signal Transduct. Target Ther. 6, 78. 10.1038/s41392-021-00486-7 33623018 PMC7902610

[B50] Zhang Y. Yao X. Zhou H. Wu X. Tian J. Zeng J. (2022). OncoSplicing: an updated database for clinically relevant alternative splicing in 33 human cancers. Nucleic Acids Res. 50, D1340–D1347. 10.1093/nar/gkab851 34554251 PMC8728274

