# Supplementary figures and images for: DASES: a database of alternative splicing for esophageal squamous cell carcinoma
Source: Front Genet. 2023 Nov 10;14:1237167. doi: 10.3389/fgene.2023.1237167 (PMC10667693; doi:10.3389/fgene.2023.1237167)

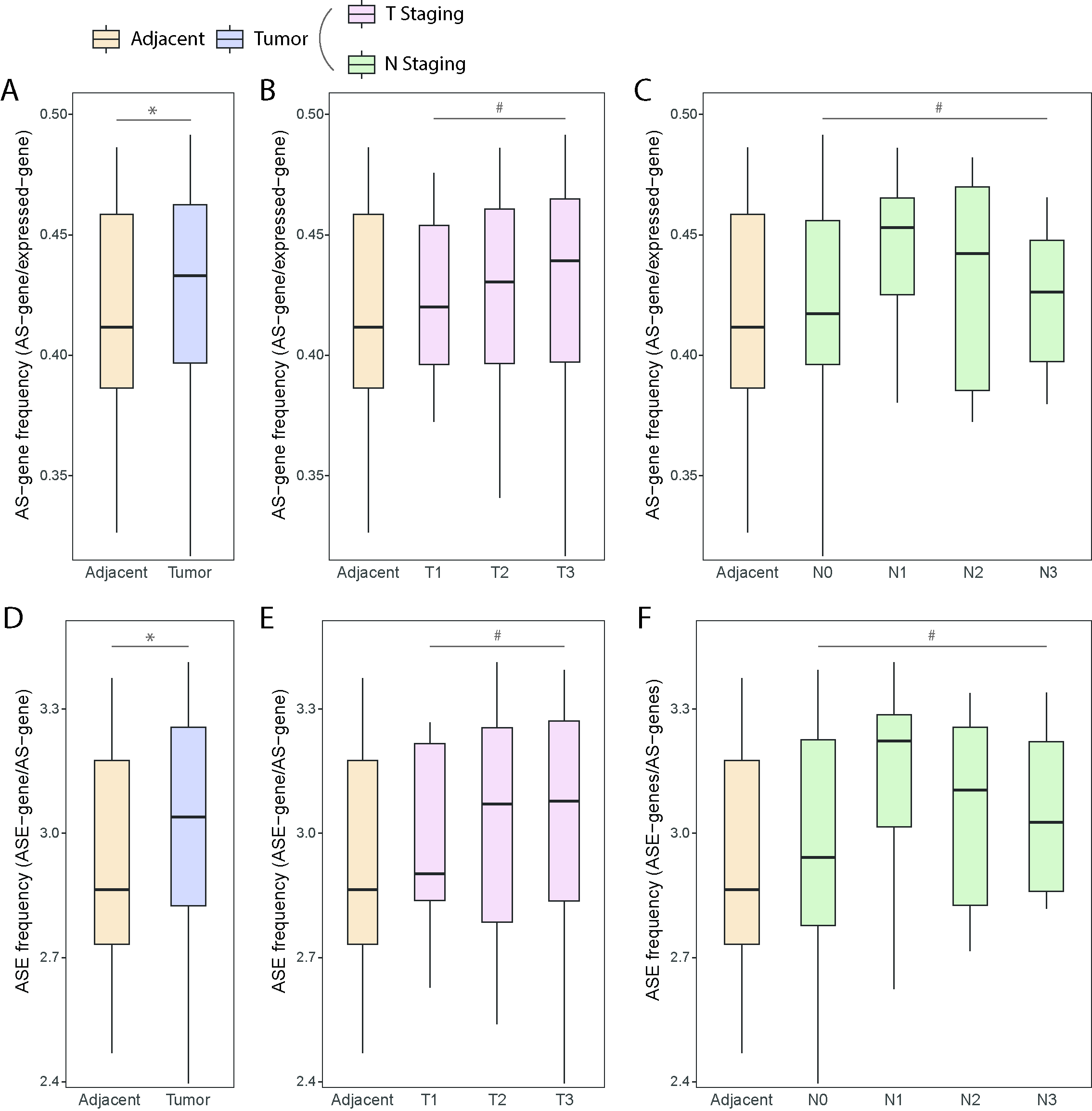

Supplement: Supplementary file 1 [file Image1.TIF]
